# Quadruple-negative autoantibody status and interstitial lung disease progression in systemic sclerosis

**DOI:** 10.1093/rap/rkag127

**Published:** 2026-10-24

**Authors:** Takeshi Kawaguchi, Kunihiko Umekita, Masayuki Murai, Chihiro Iwao, Masatoshi Kimura, Michikazu Nakai, Taiga Miyazaki

**Affiliations:** Division of Respirology, Rheumatology, Infectious Diseases and Neurology, Department of Internal Medicine, Faculty of Medicine; Division of Respirology, Rheumatology, Infectious Diseases and Neurology, Department of Internal Medicine, Faculty of Medicine; Division of Respirology, Rheumatology, Infectious Diseases and Neurology, Department of Internal Medicine, Faculty of Medicine; Division of Respirology, Rheumatology, Infectious Diseases and Neurology, Department of Internal Medicine, Faculty of Medicine; Division of Respirology, Rheumatology, Infectious Diseases and Neurology, Department of Internal Medicine, Faculty of Medicine; Department of Statistics and Data Management, Faculty of Medicine, University of Miyazaki, Miyazaki, Japan; Division of Respirology, Rheumatology, Infectious Diseases and Neurology, Department of Internal Medicine, Faculty of Medicine

**Keywords:** systemic sclerosis, interstitial lung disease, autoantibody profiles, disease progression, quadruple-negative

## Abstract

**Objectives:**

To evaluate the association between disease-specific autoantibody profiles and disease progression in systemic sclerosis-associated interstitial lung disease (SSc-ILD), with a focus on a quadruple-negative phenotype.

**Methods:**

This retrospective cohort study included 80 patients with SSc-ILD who were followed for at least 12 months at a single tertiary centre. ILD progression was defined according to the OMERACT criteria and/or worsening on high-resolution computed tomography. Autoantibody status was categorized based on four antibodies: anti-topoisomerase I, anti-centromere, anti-RNA polymerase III and anti-U1-RNP. Quadruple-negative (QN) was defined as negativity for all four antibodies. Factors associated with ILD progression were evaluated using multivariable logistic regression. Exploratory comprehensive autoantibody profiling was performed in patients with available stored serum samples.

**Results:**

During a median follow-up of 44.5 months, disease progression occurred in 32 of 80 patients with SSc-ILD. Anti-centromere antibody (ACA) positivity was associated with a lower risk of progression, whereas anti-topoisomerase I antibody positivity was not associated with disease progression (Model 2: OR 3.19, 95% CI 0.70–14.60). Compared with ACA positivity, QN status was associated with ILD progression (Model 2: OR 10.40, 95% confidence interval [CI] 1.77–60.70). However, CIs were wide. Exploratory analyses showed a high prevalence of anti-Ro-52 antibody positivity among patients with QN status.

**Conclusion:**

QN autoantibody status may be associated with an increased risk of ILD progression in SSc. The QN status may represent a heterogeneous serological category that could help identify patients who require closer clinical monitoring. However, these results are hypothesis-generating and require validation in larger, independent cohorts.

Key messages

- Quadruple-negative autoantibody status may be associated with increased risk of progression in SSc-ILD.
- Anti-centromere antibody positivity was associated with a lower risk of ILD progression.
- Routine autoantibody profiles may help identify patients with SSc-ILD requiring closer clinical monitoring.

## Introduction

Interstitial lung disease (ILD) is the most common organ manifestation of systemic sclerosis (SSc), affecting approximately 50–65% of patients as assessed by high-resolution computed tomography (HRCT) [1, 2]. Approximately 40% of patients with systemic sclerosis-associated ILD (SSc-ILD) experience disease progression, and 25–30% develop a progressive clinical phenotype that may lead to respiratory failure and death [3]. Progressive SSc-ILD is associated with poor prognosis. According to Goh *et al.* [4], patients with extensive disease, defined as ILD involving more than 20% of the lungs on HRCT and/or a predicted forced vital capacity (FVC) of less than 70%, have an approximately 50% increased risk of mortality within 5 years.

Various clinical, serological and physiological factors have been reported to predict SSc-ILD progression, including diffuse cutaneous SSc subtype, smoking history, arthritis, nucleolar antinuclear antibody pattern (reflecting anti-Th/To or anti-U3-RNP antibodies), anti-topoisomerase I antibody (ATA) positivity, elevated inflammatory markers, increased serum Krebs von den Lungen-6 (KL-6), reduced peripheral oxygen saturation during the 6-min walk test and decreased diffusing capacity for carbon monoxide (DLco) [5–11]. In addition, an accelerated decline in FVC has been observed in patients meeting the INBUILD criteria for progressive pulmonary fibrosis, including those with connective tissue disease-associated ILD, highlighting the importance of monitoring disease behaviour over time [12]. Recent prediction models and cohort studies, including SPAR, SADL and EUSTAR data, have improved risk stratification in SSc-ILD. However, many of these approaches incorporate variables reflecting already established or evolving pulmonary involvement, such as reduced DLco, oxygen desaturation and progressive disease behaviour. Conversely, autoantibody profiles are routinely available at the time of diagnosis and may provide an earlier opportunity for risk stratification.

Despite these advances, it remains challenging to identify, at the time of diagnosis, which patients with SSc-ILD are at higher risk of disease progression and may require closer follow-up. In particular, the prognostic significance of disease-specific autoantibody profiles, routinely assessed in clinical practice, has not been fully established. Therefore, this study aimed to evaluate the association between disease-specific autoantibody profiles and ILD progression in patients with SSc, with a particular focus on a quadruple-negative (QN) phenotype (i.e. negativity for anti-topoisomerase I, anti-centromere, anti-RNA polymerase III and anti-U1-RNP antibodies).

## Methods

### Study protocol

This single-centre retrospective observational study was conducted at the University of Miyazaki Hospital. Clinical and laboratory parameters at the time of diagnosis of SSc-ILD were compared between patients with and without disease progression to identify factors associated with progression.

Baseline characteristics included sex, age, smoking history, clinical symptoms, comorbidities, pulmonary function test results, serum KL-6 levels and disease-specific autoantibody status [ATA, anti-centromere antibody (ACA), anti-RNA polymerase 3 antibody (RNA-P3) and anti-U1-RNP antibody (U1-RNP)]. ATA, ACA and U1-RNP antibodies were measured via chemiluminescent enzyme immunoassays, whereas RNA-P3 antibodies were measured using enzyme-linked immunosorbent assays. Gastroesophageal reflux disease (GERD) was diagnosed based on inflammatory findings, including erosive changes, identified by upper gastrointestinal endoscopy, regardless of symptoms.

As an exploratory analysis, additional SSc-associated autoantibodies were assessed in patients with available stored serum samples using EUROLINE immunoblotting (BML, Tokyo, Japan) [13] or A-Cube multiplex protein arrays (Fushimi Pharmaceutical Co., Ltd, Kagawa, Japan) [14]. Exploratory analyses were performed using EUROLINE immunoblotting or A-Cube multiplex protein arrays due to the limited availability of stored serum samples. Autoantibodies common to both platforms (FBRL, NOR90, Th/To, PM-Scl100, PM-Scl75, Ku, and Ro-52) were analyzed.

ILD progression was defined according to the OMERACT CTD-ILD Working Group criteria and/or worsening on HRCT. According to the OMERACT definition, progression was defined as a ≥ 10% relative decline in FVC or a 5–9% relative decline in FVC combined with a ≥ 15% relative decline in DLco [15]. In addition, clinically evident worsening of ILD-related findings on HRCT was considered to indicate disease progression. HRCT assessments were performed in routine clinical practice by experienced radiologists or pulmonologists. However, formal standardized scoring or blinded central review was not applied. Individual components contributing to the composite progression outcome were not systematically recorded in the original dataset.

This study was conducted in accordance with the Declaration of Helsinki and was approved by the Research Ethics Committee of the Faculty of Medicine, University of Miyazaki (approval no. O-1416). For analyses based solely on clinical data, patient consent was obtained using an opt-out approach. For exploratory analyses involving stored serum samples, written informed consent was obtained at the time of sample collection.

### Participants

This study included adult patients aged ≥18 years who were diagnosed with SSc-ILD at the University of Miyazaki Hospital between April 2012 and June 2025. SSc was diagnosed according to the 2013 American College of Rheumatology/European League Against Rheumatism classification criteria [16]. SSc-ILD was defined as the presence of interstitial lung abnormalities on HRCT, as interpreted by experienced radiologists or pulmonologists in routine clinical practice. Given that overlapping autoimmune features are frequently observed in real-world SSc cohorts, patients with concomitant connective tissue diseases were not excluded. Patients with a follow-up period of less than 12 months were excluded. Patients who developed ILD progression within the first 12 months were included to avoid excluding early progressive cases.

### Statistical analysis

Categorical variables were expressed as numbers (percentages) and compared using Fisher’s exact test, whereas continuous variables were expressed as medians (interquartile ranges) and compared using the Mann–Whitney *U* test. Multivariable logistic regression analysis was performed to identify factors associated with SSc-ILD progression. Given the limited number of outcome events, the number of covariates included in the multivariable models was restricted in accordance with the events-per-variable principle to reduce the risk of model overfitting. Clinically relevant variables (age, sex and baseline %FVC) were selected *a priori*. For analytical purposes, patients positive for multiple autoantibodies were assigned a single dominant autoantibody according to a predefined hierarchy (ATA > RNA-P3 > ACA > U1-RNP), based on disease specificity and established clinical relevance. Patients negative for all four antibodies were classified as QN. ACA positivity was used as the reference category. Odds ratios (ORs) with 95% confidence intervals (CIs) were reported. All statistical tests were two-tailed, and a *P-*value < 0.05 was considered statistically significant.

As sensitivity analyses, we evaluated the association between QN status and ILD progression in patients with SSc alone after excluding all patients with overlapping connective tissue diseases (CTDs), and in patients with SSc with or without Sjögren’s disease (SjD) after excluding those with other overlapping CTDs. To assess the potential influence of SjD, we also performed analyses stratified by the presence or absence of SjD. Unadjusted ORs and 95% CIs were calculated using logistic regression.

Treatment variables, including immunosuppressive and antifibrotic therapies, were not included in the multivariable models because of heterogeneity in treatment regimens and limited sample size, which could have increased the risk of model overfitting.

All statistical analyses were performed using EZR (Saitama Medical Center, Jichi Medical University, Saitama, Japan), a graphical user interface for R (version 4.4.2; R Foundation for Statistical Computing, Vienna, Austria).

## Results

### Patient characteristics

This study enrolled 177 patients diagnosed with SSc at the University of Miyazaki Hospital between April 2012 and June 2025. Supplementary Table S1 shows the baseline clinical characteristics of patients with and without ILD at the time of diagnosis. SSc-ILD was diagnosed in 99 patients. Among them, 80 patients with a follow-up of at least 12 months were included in the final analysis. The patient selection process is summarized in Fig. 1.

**Figure 1:**
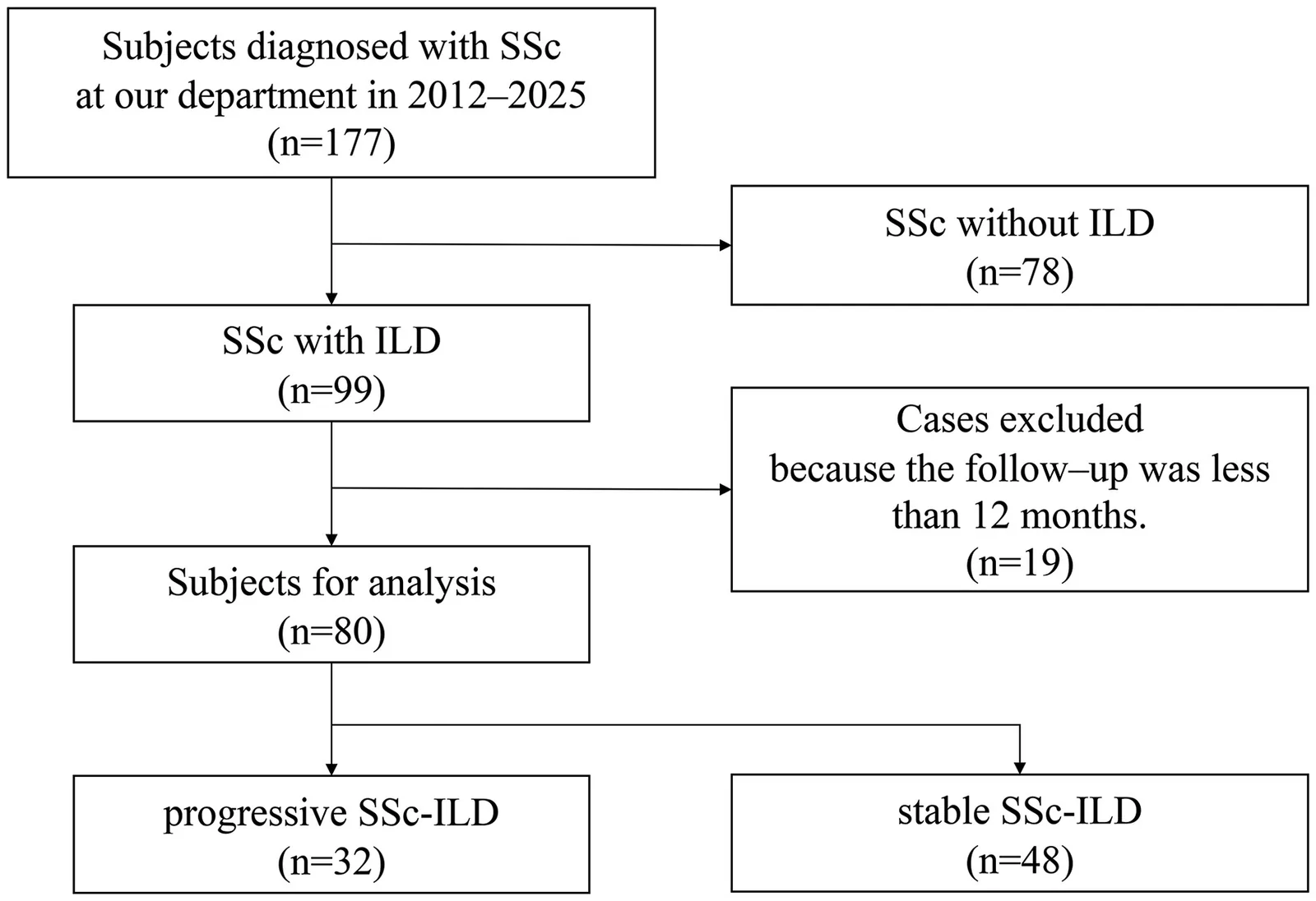
Study flow diagram. Flow diagram showing patient selection and classification into progressive and non-progressive SSc-ILD groups. SSc, systemic sclerosis; ILD, interstitial lung disease

During a median follow-up period of 44.5 months, 32 patients (40%) met the criteria for ILD progression, whereas 48 patients remained non-progressive. Their baseline clinical characteristics are shown in Table 1. Respiratory symptoms at diagnosis and extensive disease on HRCT were more frequent in patients with progressive SSc-ILD. These patients also had lower baseline %FVC and higher serum KL-6 levels. GERD was more frequently observed in patients with non-progressive disease. At the time of SSc diagnosis, most patients were untreated, except for a few patients who received low-dose corticosteroids. During follow-up, some patients received corticosteroids and immunosuppressive or antifibrotic therapies, including prednisolone (*n *= 34), intravenous cyclophosphamide (*n *= 15), azathioprine (*n *= 9), mycophenolate mofetil (*n *= 9), rituximab (*n *= 6) and nintedanib (*n *= 5).

**Table 1:** Baseline clinical characteristics of patients with SSc-ILD according to disease progression.

	SSc-ILD (*n *= 80)	Progressive (*n *= 32)	Stable (*n *= 48)	*P*-value
Males	13 (16.3)	8 (25.0)	5 (10.4)	0.122
Age at diagnosis	60.5 (49.3–68)	65 (53–71.3)	57.5 (46.8–66)	0.113
Smoking (*n *= 77)	23 (29.9)	10 (32.3)	13 (28.3)	0.801
Other CTD	38 (47.5)	16 (50)	22 (45.8)	0.820
SjD	28 (35.0)	12 (37.5)	16 (33.3)	0.812
Other CTD without SjD	17 (21.3)	6 (18.8)	11 (22.9)	0.783
Respiratory symptom	48 (60.0)	25 (78.1)	23 (47.9)	**0.010**
Diffuse cutaneous type	27 (33.8)	11 (34.4)	16 (33.3)	1
Modified Rodnan skin score (*n *= 78)	9 (4–16)	10 (4–20)	8 (3.8–13)	0.179
Raynaud’s phenomenon (*n *= 78)	71 (91.0)	25 (83.3)	46 (95.8)	0.062
Nailfold capillary abnormalities (*n *= 79)	55 (69.6)	23 (74.2)	32 (66.7)	0.442
Digital scar/ulcer (*n *= 79)	21 (26.6)	10 (32.3)	11 (22.9)	0.437
Arthritis	23 (28.8)	9 (28.1)	14 (29.2)	1
Pulmonary hypertension	21 (26.3)	11 (34.4)	10 (20.8)	0.203
GERD	27 (33.8)	5 (15.6)	22 (45.8)	**0.007**
Scleroderma renal crisis	6 (7.5)	4 (12.5)	2 (4.2)	0.211
Myositis	11 (13.8)	4 (12.5)	7 (14.6)	1
Extensive disease (*n *= 78)	34 (43.6)	20 (64.5)	14 (29.8)	**0.005**
%forced vital capacity (*n *= 78)	78.8 (68.6–97.7)	74.4 (63.4–87.7)	85.9 (70.4–107.1)	**0.029**
%DLco (*n *= 61)	71.8 (55.9–87.6)	62.9 (49.2–81.1)	75.8 (58.2–89.5)	0.084
Krebs von den Lungen-6 (*n *= 78)	555 (360.3–932.8)	774 (471.5–1296)	430 (293.5–751)	**0.010**
Anti-topoisomerase I antibody	30 (37.5)	12 (37.5)	18 (37.5)	1
Anti-centromere antibody	21 (26.3)	3 (9.4)	18 (37.5)	**0.008**
Anti-RNA polymerase III antibody	8 (10)	4 (12.5)	4 (8.3)	0.705
Anti-U1-RNP antibody	19 (23.8)	9 (28.1)	10 (20.8)	0.593
Quadruple-negative	15 (18.8)	10 (31.3)	5 (10.4)	**0.038**

Data are presented as numbers (%) or medians (interquartile ranges), unless otherwise indicated. For variables with missing data, percentages were calculated using the number of patients with available data as the denominator. Progressive SSc-ILD was defined according to the OMERACT CTD-ILD Working Group criteria and/or clinically evident worsening on HRCT. Categorical variables were compared using Fisher’s exact test, whereas continuous variables were compared using the Mann–Whitney *U* test. Extensive disease was defined as a disease extent of >20% on HRCT or 10–20% on HRCT combined with forced vital capacity < 70% predicted. Statistically significant *P*-values (*P* < 0.05) are shown in bold. Abbreviations: anti-U1-RNP, anti-U1 ribonucleoprotein; CTD, connective tissue diseases; DLco, diffusing capacity of the lung for carbon monoxide; GERD, gastroesophageal reflux disease; ILD, interstitial lung disease; SjD, Sjögren’s disease; SSc, systemic sclerosis.

Regarding disease-specific autoantibodies, ACA positivity was less frequent among patients with progressive disease, whereas QN status was more frequent in this group. The frequency of ATA positivity did not differ between patients with progressive and non-progressive disease. Multiple disease-specific autoantibody positivity was observed: ATA plus U1-RNP in 8 patients, ATA plus RNA-P3 in 1 patient, and ACA plus U1-RNP in 4 patients. A total of 19 patients were positive for U1-RNP. Among them, 8 patients had isolated U1-RNP positivity.

### Sensitivity analyses

In sensitivity analyses, the effect estimates for QN status were in the same direction as the primary analysis. Among patients with SSc with or without SjD (*n* = 63), QN status was associated with ILD progression (OR 6.45, 95% CI 1.22–34.2). Among patients with SSc alone (*n* = 42), the effect estimate remained similar in direction and magnitude, although the confidence interval was wide (OR 4.00, 95% CI 0.64–25.0) (Supplementary Table S2, available at *Rheumatology Advances in Practice* Online). In analyses stratified by SjD status, effect estimates were similar in patients with SjD (OR 3.50, 95% CI 0.52–23.60) and those without SjD (OR 4.14, 95% CI 0.90–19.00), although the confidence intervals were wide and neither estimate reached statistical significance (Supplementary Table S3, available at *Rheumatology Advances in Practice* Online).

### Multivariable analysis

The results of the multivariable logistic regression analyses are presented in Table 2. After adjustment for age and sex (Model 1), QN status was associated with ILD progression compared with ACA positivity, whereas ACA positivity was associated with a lower risk of progression. ATA positivity was not associated with ILD progression. These findings were generally consistent after additional adjustment for baseline %FVC (Model 2). Given the limited sample size and number of outcome events, the estimated effect sizes should be interpreted with caution.

**Table 2:** Multivariable logistic regression analyses of factors associated with SSc-ILD progression.

Variables	Model 1 OR (95% CI)	*P*-value	Model 2 OR (95% CI)	*P*-value
Quadruple-negative	13.71 (2.45–76.70)	**0.003**	10.40 (1.77–60.70)	**0.01**
Anti-topoisomerase I antibody	4.61 (1.07–19.90)	**0.04**	3.19 (0.70–14.60)	0.14
Anti-U1-RNP antibody	8.59 (1.06–69.5)	**0.04**	7.58 (0.87–66.10)	0.07
Anti-RNA polymerase III antibody	6.34 (0.86–46.74)	0.07	7.88 (0.90–69.10)	0.06
Age (per year)	1.02 (0.99–1.08)	0.06	1.04 (1.00–1.09)	0.05
Male sex	2.20 (0.53–9.20)	0.28	2.94 (0.67–13.00)	0.16
%forced vital capacity (per 1%)	—	—	0.97 (0.95–1.00)	0.05

ORs and 95% CIs were estimated using multivariable logistic regression models. Anti-centromere antibody was used as the reference category for comparisons among autoantibody groups. Model 1 was adjusted for age and sex. Model 2 was additionally adjusted for baseline %forced vital capacity. Statistically significant *P*-values (*P* < 0.05) are shown in bold. Abbreviations: anti-U1-RNP, anti-U1 ribonucleoprotein; CI, confidence interval; ILD, interstitial lung disease; OR, odds ratio; SSc, systemic sclerosis.

### Exploratory autoantibody analysis

An exploratory analysis of additional SSc-associated autoantibodies was performed in a subset of 51 patients with available stored serum samples. Anti-Ro-52 antibody positivity was frequently observed among patients with QN status based on routine disease-specific autoantibody testing (Fig. 2A). Comprehensive autoantibody testing was performed in 11 patients with QN status, among whom anti-Th/To antibodies were detected in two patients, whereas none were positive for FBRL, NOR90, PM-Scl100, PM-Scl75 or Ku antibodies.

**Figure 2:**
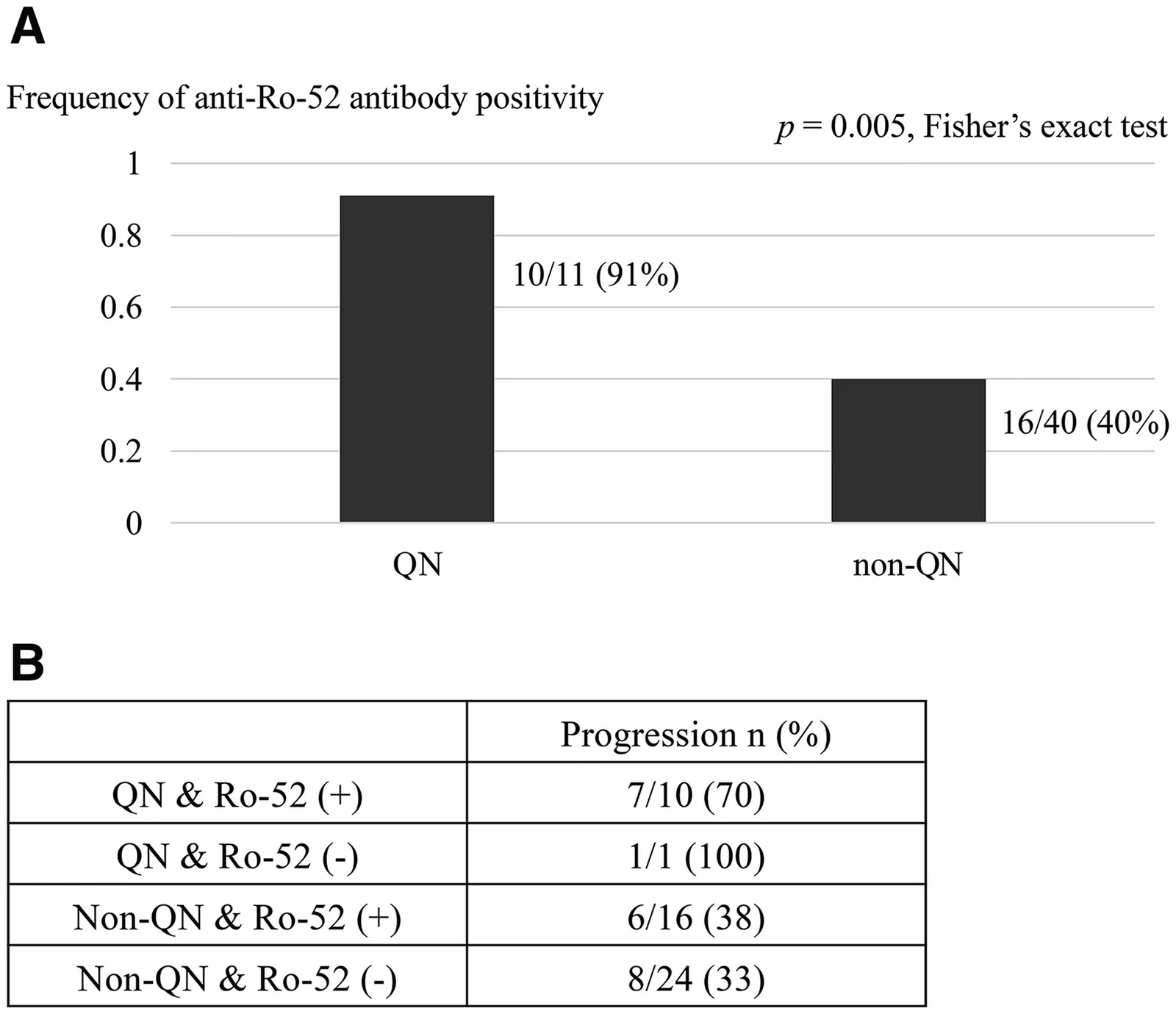
Exploratory analysis of anti-Ro-52 antibody status in quadruple-negative systemic sclerosis-associated interstitial lung disease. (A) Frequency of anti-Ro-52 antibody positivity in patients with QN and non-QN status among patients with available serum samples. (B) Proportion of progressive interstitial lung disease according to QN and anti-Ro-52 antibody status. As the subgroup analyses were exploratory and limited by small sample sizes, the findings should be interpreted with caution. QN, quadruple-negative

When patients were stratified by QN status and anti-Ro-52 antibody positivity, ILD progression appeared to be more frequent in patients with QN status, regardless of anti-Ro-52 status (Fig. 2B). However, due to the limited sample size, particularly in subgroup analyses, no formal statistical comparisons were performed.

## Discussion

This retrospective cohort study of patients with SSc-ILD suggests that QN autoantibody status may be associated with ILD progression, whereas ATA positivity was not clearly associated with disease progression. By incorporating U1-RNP status and focusing on clinically relevant disease progression, these findings may provide additional insight into prognostic stratification in SSc-ILD. Previous studies have defined antibody-negative subsets based on three autoantibodies [17, 18], whereas the present study included U1-RNP status, which accounted for 23.8% of patients with SSc-ILD in our cohort. This approach can help identify a broader and potentially heterogeneous serological category, including patients without routinely detected disease-specific autoantibodies. Meanwhile, the inverse association observed between GERD and ILD progression may reflect confounding by disease phenotype, such as ACA positivity, rather than a direct protective effect. Although most patients with GERD were likely treated with proton pump inhibitors or potassium-competitive acid blockers in routine clinical practice, detailed data on acid-suppressive therapy were not systematically collected. Treatment exposure during follow-up was heterogeneous and might have influenced disease progression. Furthermore, the cohort included patients diagnosed before the approval of mycophenolate mofetil and nintedanib in Japan, thereby leading to differences in treatment strategies across time periods. Given the limited sample size and time-varying nature of treatment exposure, treatment variables were not included in the multivariable models.

Sensitivity analyses yielded effect estimates in the same direction as the primary analysis. In analyses stratified by SjD status, the effect estimates were similar in direction and magnitude in patients with and without SjD, although the confidence intervals were wide and neither estimate reached statistical significance. These findings do not suggest that the observed association was driven primarily by the presence of SjD; however, the limited sample sizes preclude definitive conclusions. Given the frequent coexistence of SjD in patients with SSc, the analysis including patients with SSc with or without SjD may better reflect the heterogeneity encountered in clinical practice. In contrast, the analysis restricted to patients without overlapping CTDs included approximately half of the original cohort, resulting in reduced statistical precision, and should therefore be interpreted with caution.

ACA was used as the reference category because it has been consistently associated with a lower risk of ILD and slower disease progression in SSc. Previous studies have reported that ATA positivity may be associated with the presence and severity of SSc-ILD [10], although its role in predicting disease progression remains uncertain. In the SENSCIS trial, ATA positivity was not associated with a greater decline in FVC after adjustment for clinical factors [19]. In line with these observations, ATA positivity was not associated with ILD progression in our analysis. These findings suggest that ATA may reflect susceptibility to ILD development rather than subsequent disease behaviour. In contrast, patients with QN status may represent a subgroup at an increased risk of disease progression, identifiable using routinely available serological markers.

While previous studies have primarily focused on triple-negative autoantibody status, the present study evaluates a broader serological definition incorporating four routinely tested autoantibodies, including U1-RNP. Several studies have reported an association between triple-negative status and SSc-ILD. For example, Kruzer *et al.* [17] reported that approximately 14% of patients with ANA-positive SSc had triple-negative disease, among whom ILD was present in 60%, suggesting a substantial burden of ILD in this subgroup. In addition, a recent post hoc analysis of the SENSCIS trial suggested an association between triple-negative status and SSc-ILD progression [18]. In this context, our findings may extend these observations by suggesting that quadruple-negative autoantibody status may also be associated with clinically relevant ILD progression in routine clinical practice. Importantly, in addition to decline in %FVC, the present study evaluated composite progression based on OMERACT criteria, which may better capture clinically meaningful worsening in real-world settings. However, the use of both OMERACT criteria and radiographic worsening on HRCT may have introduced heterogeneity into outcome assessment.

QN status in SSc may reflect a subgroup not fully captured by conventional disease-specific autoantibodies, potentially characterized by non-classical autoantibody profiles. In our exploratory analysis, anti-Ro-52 antibody positivity was frequently observed among patients with QN status. Similarly, Kruzer *et al.* [17] reported that anti-Ro-52 was the most prevalent antibody in triple-negative SSc. Although comprehensive profiling was not performed in all cases, anti-Ro-52 antibodies were more frequently detected in QN than in non-QN patients in our cohort. Previous studies have also suggested a potential association between anti-Ro-52 antibodies and adverse pulmonary outcomes in SSc [20, 21]. These findings indicate that the QN status should not be interpreted as true seronegativity but may instead reflect a non-classical autoantibody profile. Further studies are needed to clarify the autoantibody profiles of patients with triple-negative and quadruple-negative SSc and to determine whether anti-Ro-52 antibody positivity contributes to SSc-ILD progression. Although the mechanism remains unclear, recent Japanese cohort studies have shown that non-classical autoantibody profiles, including anti-SSA and anti-Ro-52 antibody positivity, may be associated with adverse pulmonary involvement and disease progression in SSc [22, 23]. These observations may reflect underlying heterogeneity in immune phenotypes among patients with QN status. Clinically, identification of QN status at the time of SSc-ILD diagnosis may help recognize patients who require closer monitoring, even in the absence of classical high-risk autoantibodies. These findings also suggest that reliance on ATA status alone may be insufficient for prognostic assessment in SSc-ILD.

This study has several limitations. First, its retrospective design and Japanese single-centre setting may limit the generalizability of the findings. Differences in clinical characteristics, autoantibody distributions and treatment strategies across ethnicities and healthcare systems may limit the applicability of our findings to European and multinational cohorts. In addition, given the relatively small sample size and limited number of outcome events, caution should be exercised when interpreting the effect estimates because of considerable uncertainty and potential model instability. Furthermore, because of the small number of overlapping autoantibody combinations, sensitivity analyses could not be performed without hierarchical assignment. Sensitivity analyses excluding patients with overlapping CTDs were performed; however, the reduced sample size, particularly in the analysis restricted to patients without overlapping CTDs, limits the precision and interpretation of these findings. Therefore, the findings should be considered hypothesis-generating. The number of outcome events relative to the number of covariates may have introduced model overfitting and unstable estimates, although efforts were made to limit the number of variables included in the multivariable models. Residual confounding cannot be excluded. Treatment variables were not included in the multivariable analyses because of heterogeneity in treatment regimens and the limited sample size, which may have increased the risk of model overfitting and may have reduced the reliability of the estimates. Second, the timing and frequency of pulmonary function tests and HRCT assessments were not standardized, which may have influenced the evaluation of disease progression. In addition, HRCT assessments were based on routine clinical evaluation without standardized scoring or blinded central review, which may have introduced variability in outcome assessment. Because the individual components of the composite progression outcome were not systematically captured, the proportion of patients meeting the OMERACT criteria alone, HRCT worsening alone or both could not be determined. Therefore, an OMERACT-only sensitivity analysis could not be performed, which may have introduced heterogeneity and potential measurement bias in outcome classification. Third, only a subset of patients underwent comprehensive autoantibody profiling, limiting the sample size for exploratory analyses. In addition, exploratory autoantibody profiling was performed using two assay platforms with different analytical characteristics, and formal concordance between assays was not evaluated. Therefore, findings related to additional autoantibodies, including anti-Ro-52 and anti-Th/To antibodies, should be interpreted with caution. In particular, the subgroup sizes in the exploratory analyses were very small, thereby limiting statistical robustness and precluding definitive conclusions. Therefore, external validation in larger multicentre, multi-ethnic cohorts is warranted.

In conclusion, this study provides clinically relevant insights into the potential role of routinely assessed autoantibody profiles in the risk stratification of SSc-ILD. In particular, QN autoantibody status may be associated with ILD progression, whereas ATA positivity was not clearly associated with disease progression. These findings suggest that the absence of established disease-specific autoantibodies may help identify patients at increased risk of disease progression and require closer clinical monitoring. Such patients may benefit from more careful longitudinal assessment, including closer surveillance of pulmonary function and HRCT findings. However, these findings should be considered hypothesis-generating and require validation in larger, independent cohorts.

## Supplementary Material

rkag127_Supplementary_Data

## Data Availability

The data underlying this article will be shared on reasonable request to the corresponding author.
