# Supplementary material for: Quadruple-negative autoantibody status and interstitial lung disease progression in systemic sclerosis
Source: Rheumatol Adv Pract. 2026 Oct 24;10(4):rkag127. doi: 10.1093/rap/rkag127 (PMC13760280; doi:10.1093/rap/rkag127)
Supplement: Supplementary file 1 [file rkag127_supplementary_data.zip › Figure_2B.pdf]

	Progression n (%)
QN & Ro-52 (+)	7/10 (70)
QN & Ro-52 (-)	1/1 (100)
Non-QN & Ro-52 (+)	6/16 (38)
Non-QN & Ro-52 (-)	8/24 (33)
