# Supplementary material for: Quadruple-negative autoantibody status and interstitial lung disease progression in systemic sclerosis
Source: Rheumatol Adv Pract. 2026 Oct 24;10(4):rkag127. doi: 10.1093/rap/rkag127 (PMC13760280; doi:10.1093/rap/rkag127)
Supplement: Supplementary file 1 [file rkag127_supplementary_data.zip › Figure_1.pdf]

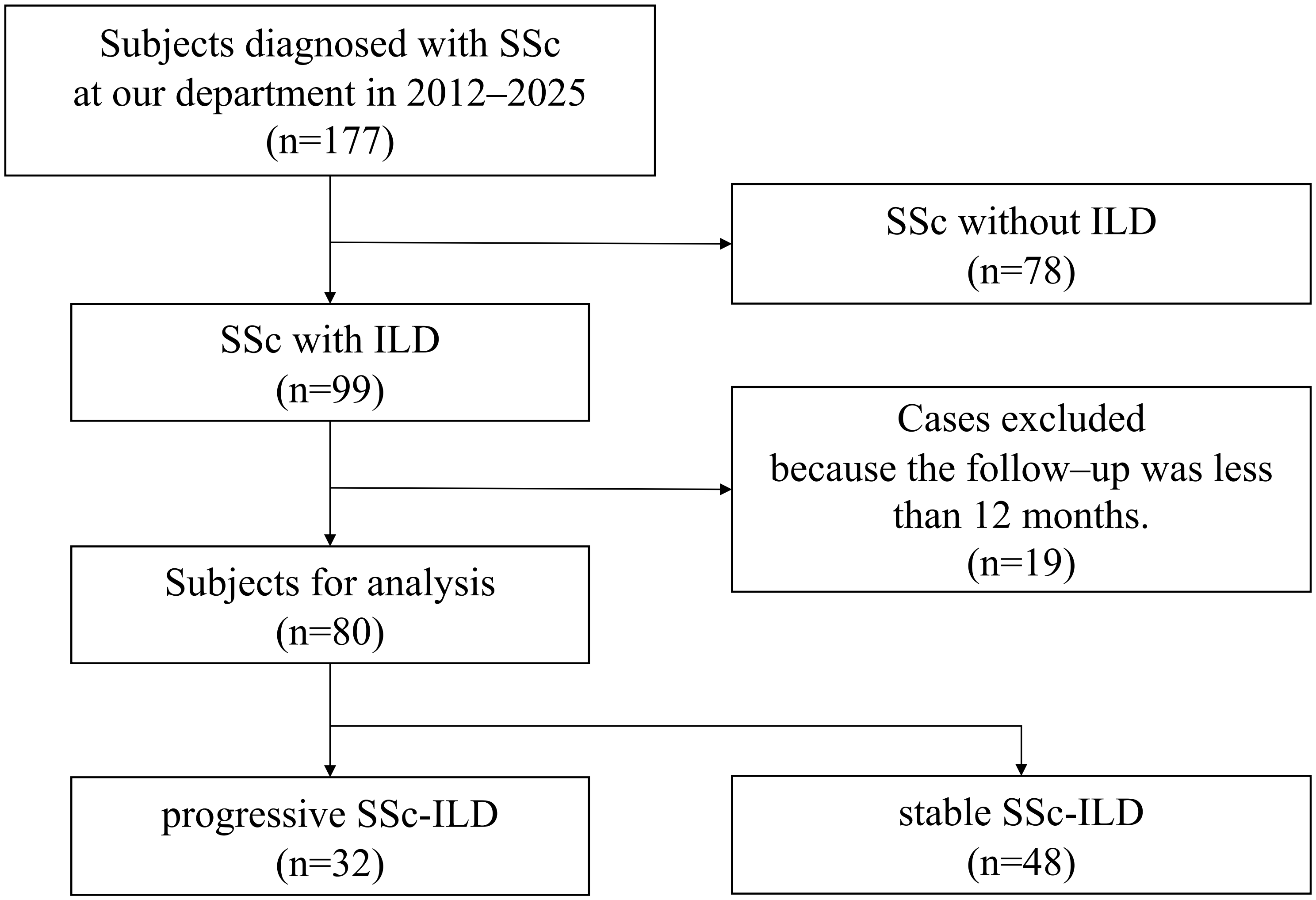
Subjects diagnosed with SSc
at our department in 2012–2025
(n=177)
SSc without ILD
(n=78)
SSc with ILD
(n=99)
Cases excluded
because the follow–up was less
than 12 months.
(n=19)
Subjects for analysis
(n=80)
progressive SSc-ILD
(n=32)
stable SSc-ILD
(n=48)
