# Supplementary material for: Quadruple-negative autoantibody status and interstitial lung disease progression in systemic sclerosis
Source: Rheumatol Adv Pract. 2026 Oct 24;10(4):rkag127. doi: 10.1093/rap/rkag127 (PMC13760280; doi:10.1093/rap/rkag127)
Supplement: Supplementary file 1 [file rkag127_supplementary_data.zip › Figure_2A.pdf]

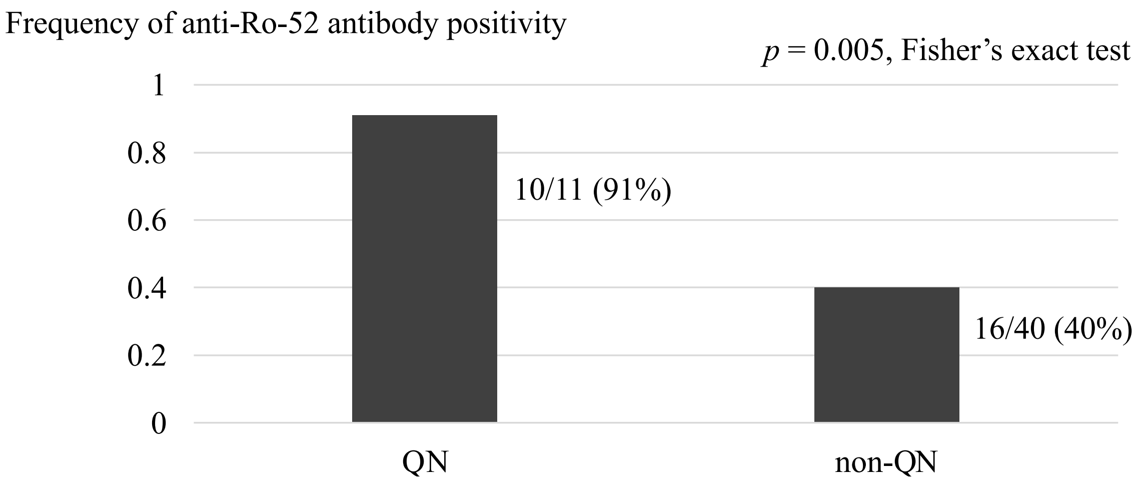

Frequency of anti-Ro-52 antibody positivity
p = 0.005, Fisher's exact test
1
0.8
0.6
0.4
0.2
0
10/11 (91%)
16/40 (40%)
QN
non-QN
