# Supplementary material for: Quadruple-negative autoantibody status and interstitial lung disease progression in systemic sclerosis
Source: Rheumatol Adv Pract. 2026 Oct 24;10(4):rkag127. doi: 10.1093/rap/rkag127 (PMC13760280; doi:10.1093/rap/rkag127)
Supplement: Supplementary file 1 [file rkag127_supplementary_data.zip › Table S1.docx]

Supplementary Table S1. Baseline clinical characteristics of patients with systemic sclerosis with and without interstitial lung disease

|  | SSc  (n = 177) | SSc-ILD  (n = 99) | SSc without ILD  (n = 78) | p value |
| --- | --- | --- | --- | --- |
| Male subjects | 26 (14.7) | 17 (17.2) | 9 (11.5) | 0.393 |
| Age at diagnosis | 61 [50, 70] | 62 [50.5, 68] | 61 [50.3, 72] | 0.462 |
| Smoking (n=170) | 52 (29.4) | 31 (31.3) | 21 (26.9) | 0.618 |
| Other connective tissue diseases | 84 (47.5) | 49 (49.5) | 35 (44.9) | 0.549 |
| Sjogren disease | 71 (40.1) | 38 (38.4) | 33 (42.3) | 0.644 |
| Other CTD without SjD | 30 (16.9) | 20 (20.2) | 10 (12.8) | 0.229 |
| Respiratory symptom | 70 (39.5) | 63 (63.6) | 7 (9) | **<0.001** |
| Diffuse cutaneous type | 42 (23.7) | 32 (32.3) | 10 (12.8) | **0.002** |
| modified Rodnan skin score (n=172) | 8 [3, 14] | 8.5 [3.8, 16] | 6 [3, 12] | 0.108 |
| Raynaud's phenomenon (n=175) | 156 (88.1) | 89 (89.9) | 67 (85.9) | 0.275 |
| Nailfold capillary abnormalities (n=173) | 120 (67.8) | 66 (66.7) | 54 (69.2) | 0.704 |
| Digital scar/ulcer (n=176) | 44 (24.9) | 24 (24.2) | 20 (25.6) | 0.863 |
| Arthritis | 37 (20.9) | 28 (28.3) | 9 (11.5) | **0.008** |
| Pulmonary hypertension | 32 (18.1) | 23 (23.2) | 9 (11.5) | 0.051 |
| gastroesophageal reflux disease | 60 (33.9) | 33 (33.3) | 27 (34.6) | 0.873 |
| scleroderma renal crisis | 10 (5.6) | 7 (7.1) | 3 (3.8) | 0.516 |
| Myositis | 14 (7.9) | 12 (12.1) | 2 (2.6) | 0.024 |
| extensive disease (n=172) |  | 41 (41.4) |  |  |
| %forced vital capacity (n=163) | 90.8 [72.8, 106.9] | 79 [68.6, 102.8] | 101.9 [89.3, 109.6] | **<0.001** |
| %DLco (n=139) | 79.7 [63.9, 93.1] | 72.6 [57, 87.9] | 85.7 [67.4, 102.6] | **<0.001** |
| Krebs von den Lungen-6 (n=161) | 369 [207, 671] | 558 [364, 875] | 211.5 [172.3, 311.5] | **<0.001** |
| anti–topoisomerase Ⅰ antibody | 41 (23.2) | 34 (34.3) | 7 (9) | **<0.001** |
| anti-centromere antibody | 89 (50.3) | 28 (28.3) | 61 (78.2) | **<0.001** |
| anti–RNA polymerase III antibody | 17 (9.6) | 12 (12.1) | 5 (6.4) | 0.305 |
| anti–U1 ribonucleoprotein antibody | 31 (17.5) | 22 (22.2) | 9 (11.5) | 0.074 |
| Quadruple-negative | 22 (12.4) | 18 (18.2) | 4 (5.1) | **0.011** |

Values are presented as number (%) or median [interquartile range], unless otherwise indicated. Categorical variables were compared using Fisher’s exact test and continuous variables were compared using the Mann–Whitney U test. Extensive disease was defined as >20% disease extent on high-resolution computed tomography (HRCT) or 10%–20% disease extent on HRCT combined with forced vital capacity <70% predicted. CTD, connective tissue disease; DLco, diffusing capacity of the lung for carbon monoxide; ILD, interstitial lung disease; SjD, Sjögren’s disease; SSc, systemic sclerosis.

Supplementary Table S2. Supplementary Table S2. Sensitivity analyses of the association between quadruple-negative status and ILD progression after exclusion of patients with overlapping connective tissue diseases

| Cohort | n | Progressive ILD: QN | Progressive ILD: non-QN | Unadjusted OR  (95% CI) | *P*-value |
| --- | --- | --- | --- | --- | --- |
| Overall SSc cohort | 80 | 10/15 (66.7%) | 22/65 (33.8%) | 3.91 (1.19–12.90) | **0.025** |
| SSc with or without SjD | 63 | 7/9 (77.8%) | 19/54 (35.2%) | 6.45 (1.22–34.20) | 0.029 |
| SSc without overlapping CTDs | 42 | 4/6 (66.7%) | 12/36 (33.3%) | 4.00 (0.64–25.00) | 0.138 |

Unadjusted odds ratios and 95% confidence intervals were estimated using univariable logistic regression. The SSc with or without SjD cohort excluded patients with overlapping CTDs other than SjD, whereas the SSc without overlapping CTDs cohort excluded all patients with concomitant CTDs. CI, confidence interval; CTD, connective tissue disease; ILD, interstitial lung disease; OR, odds ratio; QN, quadruple-negative; SjD, Sjögren’s disease; SSc, systemic sclerosis.
